# Mesenchymal stem-cell potential in cartilage repair: an update

**DOI:** 10.1111/jcmm.12378

**Published:** 2014-10-29

**Authors:** M Mazor, E Lespessailles, R Coursier, R Daniellou, T M Best, H Toumi

**Affiliations:** a IPROS, CHRO, EA4708 Orleans UniversityOrleans, France; b Groupement des Hôpitaux de l'Institut Catholique de Lillie (GHICL)/Faculté Libre de MédecineLille, France; c Département de traumatologie-orthopédie France, UCLilleLille, France; d CNRS, ICOA, UMR 7311, Université OrléansOrleans, France; e Division of Sports Medicine, Department of Family Medicine, Sports Health And Performance Institute, The Ohio State UniversityColumbus, OH, USA

**Keywords:** osteoarthritis, cartilage, repair, mesenchymal stem cell, growth factors

## Abstract

Articular cartilage damage and subsequent degeneration are a frequent occurrence in synovial joints. Treatment of these lesions is a challenge because this tissue is incapable of quality repair and/or regeneration to its native state. Non-operative treatments endeavour to control symptoms and include anti-inflammatory medications, viscosupplementation, bracing, orthotics and activity modification. Classical surgical techniques for articular cartilage lesions are frequently insufficient in restoring normal anatomy and function and in many cases, it has not been possible to achieve the desired results. Consequently, researchers and clinicians are focusing on alternative methods for cartilage preservation and repair. Recently, cell-based therapy has become a key focus of tissue engineering research to achieve functional replacement of articular cartilage. The present manuscript is a brief review of stem cells and their potential in the treatment of early OA (*i.e*. articular cartilage pathology) and recent progress in the field.

- Introduction
- Osteoarthritis
  – What happens to OA cartilage?
  – Molecular changes in OA cartilage
- Stem-cell potential in OA
  – MSC potential for cartilage repair
  – MSC regulation
- Chondroprogenitor cells
  – Chondroprogenitor potential
  – Chondrogenesis
- Role of growth factors in cartilage repair
- Conclusion

## Introduction

Osteoarthritis (OA) is an active pathological process and is the most common degenerative orthopaedic disease [1,2]. The current view holds that OA involves not only the articular cartilage but the entire joint organ including the subchondral bone and the synovium. However, articular cartilage breakdown remains the principal characteristic of OA. Cartilage self-renewal potential is limited and consequently, the progression of degradation leads to destruction of cartilage and development of OA. Unfortunately, OA is generally diagnosed in more advanced stages, when pain and restriction of morbidity arise and clinical and radiographic signs become evident [3]. Despite drugs used clinically to reduce pain and maintain joint movement, in many cases, surgical substitution with artificial implants is inevitable. There are also a number of surgical treatment strategies currently available for articular cartilage defect repair including abrasion chondroplasty, subchondral drilling, microfracture and mosaicplasty. However, to date, these techniques have shown variable results.

It has been consistently demonstrated that cartilage defects extending to the subchondral bone do exhibit some ability to repair through formation of neocartilage, probably as a result of the release of bone marrow-derived stem cells, from the underlying subchondral bone [4,5]. This fact, coupled with the multilineage capacity potential of mesenchymal stem cells (MSC) from different sources, has made them widely investigated and utilized in cartilage repair. Tissue engineering strategies combining cell therapy (*e.g*. chondrocytes and adult stem cells) with proper biomaterials of natural or synthetic origin as scaffolds, as well as various growth and differentiation stimuli, have also been considered as a promising new approach for the treatment of articular cartilage defects. Substantial efforts have been made to choose appropriate cell sources as well as the proper growth factors and scaffolds to mimic the natural cartilage microenvironment. The main purpose of the present review was to examine the current status of stem cells in cartilage preservation/repair with respect to their potential application in orthopaedic surgery.

## Osteoarthritis

Osteoarthritis is a chronic disease involving progressive degeneration of the articular cartilage and sub-chondral bone along with synovitis [6]. Articular cartilage degeneration often occurs in response to inappropriate mechanical stress and low-grade systemic inflammation associated with trauma, obesity, sex and genetic predisposition [7,8]. It commonly occurs in the weight-bearing joints of the hips, knees and spine, but also the fingers, neck and shoulder [9]. Other joints might be also affected if prior injury or excessive mechanical stress occurs [9].

Clinical indicators of OA include joint pain, stiffness, movement limitation, crepitus, effusion and varying degrees of inflammation [6]. While the cause of primary OA is largely unknown, secondary OA is often because of trauma, acute or recurrent dislocation, or prior surgery. The major problem in diagnosing OA in middle-aged patients is that those with OA symptoms (*i.e*. pain, swelling, or stiffness) do not always express signs of the disease on X-rays or even MRIs. The opposite is also true: Those with radiological evidence of the disease (*i.e*. joint narrowing and osteophytes) are not always symptomatic. Osteoarthritis affects over 28 million people in the United States, resulting in over 50% of total joint replacements, and costing more than USD15 billion per year [10]. To provide data on the prevalence, epidemiology and aetiology of OA, Widuchowski *et al*. reviewed 25,124 knee arthroscopies and found cartilage lesions in 60% of these patients [11].

Characteristic morphological features of OA are variable including phenotypic changes in cartilage cells, progressive fibrillation of articular cartilage, subchondral bone sclerosis, osteophyte formation and increased remodelling of the periarticular bone [12].

### What happens to OA cartilage?

Adult articular cartilage contains a relatively sparse population of non-proliferating chondrocytes corresponding to 5% of the tissue's wet weight [13]. Chondrocytes are embedded within an extracellular matrix (ECM) [14]. The ECM contains mainly water and electrolytes that are bound to collagens (types II, IX and XI) and proteoglycans (aggrecan) [14]. Articular cartilage mechanical behaviour is determined by the fluid–solid interaction [15]. Proteoglycans are constrained within the collagen fibrillar network and form the extrafibrillar matrix [15]. Mechanical pressure on the joint induces compression of the articular cartilage, which raises the excursion of water, thus increasing the concentration of ions within the tissue, leading to swelling pressures [16]. The swelling pressure within the ECM is determined by the difference in ion concentration between the inside and the outside [16]. Such pressure balance could be altered when the amount of water in the ECM increases as a result of collagen network degradation typically seen in OA [16,17].

The ECM of hyaline cartilage is organized into four distinct zones (‘the superficial tangential zone, the middle (or transitional) zone, the deep (or radial) zone and the calcified zone’), with varying biochemical compositions throughout the cartilage [13]. Cartilage tissue is avascular and aneural [14]. Nutrient and gas exchange takes place through diffusion from capillaries in adjacent connective tissue (perichondrium) or through synovial fluid from joint cavities [8,12–14,18,19]. Cartilage structure and function are supported by a complex molecular backdrop of growth factors, cytokines, enzymes and transcription factors necessary for maintenance of tissue homoeostasis [2,7,12,20]. During development of OA, changes occur in metabolic activity and the homeostasis of the tissue is disturbed, resulting in a mismatch of anabolic and catabolic processes [2]. Prevalence of catabolic processes results in degradation of the ECM and subsequent cartilage destruction [2]. Over time, cartilage may degenerate at the surface which in turn progresses to the deeper areas and reaches the subchondral plate resulting in cyst and osteophyte formation [6].

### Molecular changes in OA cartilage

**Fig. 1:**
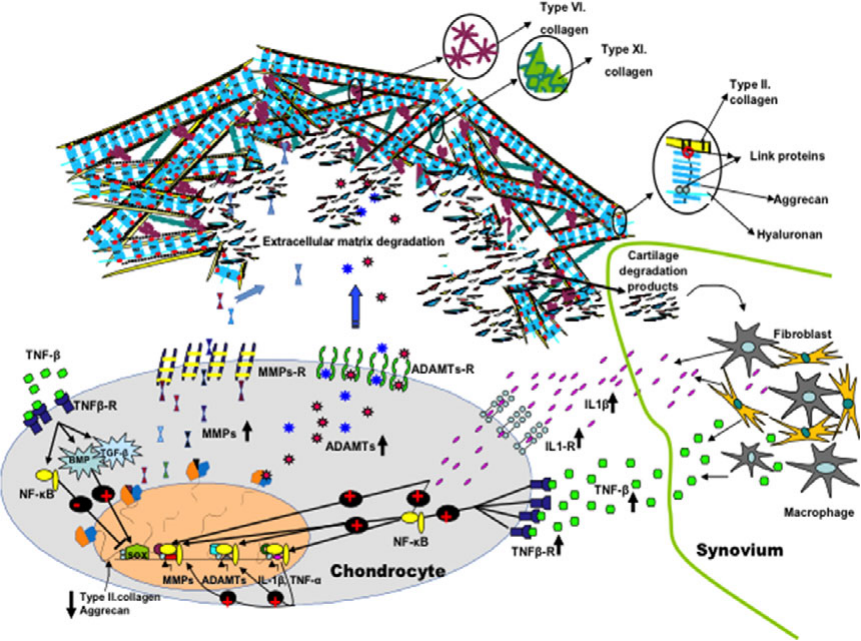
Molecular changes in osteoarthritic (OA) cartilage. The main matrix-degrading enzymes are matrix metalloproteinases (MMPs). MMPs are up-regulated in OA and included in the over-degeneration of a main extracellular matrix components: types II, VI, XI collagens and proteoglycans. At early stages, the degradation of the predominant proteoglycan, aggrecan is mainly caused by aggrecanases, (ADAMTS). The expression of matrix-degrading enzymes is further fuelled by the appearance of inflammation. Once degraded, cartilage fragments fall into the joint and contact the synovium. In contact with foreign bodies, synovial cells react by producing inflammatory mediators (IL-1β and TNF-β), which leads to additional activation of MMPs, cytokines and further cartilage degradation. Yet, positive feedback such as the activation of the bone morphogenetic proteins (BMPs) and tumour growth factor-β (TGF-β) under the control of IL-1β and TNF-β contribution in maintaining matrix synthesis. It is well known that one of the key transcription factors (SOX9) indebted in expression of collagen type II and aggrecan is regulated by BMPs and TGF-β. On the other hand, negative regulator of SOX9 expression is NF-κ B and mainly regulated by TNF-α and IL-1β.

**Fig. 2:**
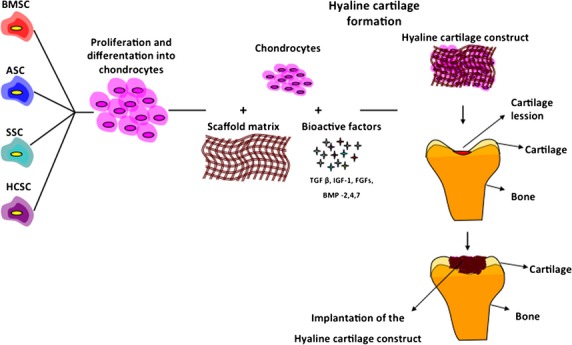
Cell-based repair of cartilage lesions. MSCs isolated from various tissues have the potential to undergo chondrogenesis and form hyaline cartilage. Furthermore, to form hyaline cartilage tissue, chondrocytes combine with appropriate scaffold matrix and bioactive factors to promote ECM formation. Constructed hyaline cartilage tissue is then implanted in the cartilage lesion site. BMCS, bone marrow-derived stem cells; ASC, adipose-derived stem cells; SSC, synovium-derived stem cells; HCSC, hyaline cartilage-derived stem cells.

In the absence of joint damage and disease, the quiescent chondrocytes maintain a low turnover replacement rate of cartilage matrix proteins [21]. With onset of OA, chondrocyte proliferation begins with cluster formation accompanied by prevalence of catabolic activity of matrix-degrading enzymes over anabolic activity of matrix proteins [2,22,23]. The expression of matrix-degrading enzymes is further fueled by an increase in growth factor expression and the appearance of inflammation [2,22–24]. The main matrix-degrading enzymes are the matrix metalloproteinases (MMPs), a family of nine or more highly homologous Zn (++)-endopeptidases that collectively cleave most of the ECM constituents [25]. It is well known that under normal conditions, these enzymes are expressed at a low level in both chondrocytes and synovial cells [9]. However, OA cartilage shows an increase in the amount of MMP-2, MMP-3, MMP-8, MMP-9, MMP-13 and MMP-14, which are included in the degradation of a wide spectra of substrates: types I, II, III, V, VI, VII, X and XI collagens, fibronectin, gelatin, elastin and proteoglycans. These are the main components, which maintain articular cartilage integrity [21,25–31]. Both human and animal studies have shown MMP-13 to be a dominant factor in collagen type II degradation [32]. The abnormal expression of MMP-13 was demonstrated by an amended epigenetic profile with up growth of 4% to 20% in non-methylated sites in normal *versus* OA chondrocytes [33]. Furthermore, microarray and RT-PCR data have highlighted MMP-13 as a major collagenase with moderate expression in early stages of OA, but overexpressed in advanced stages of the disease [33,34]. In animal studies, postnatal constitutive expression of *MMP-13* pathological changes was demonstrated to be similar to that seen in humans by loss of proteoglycans and cleavage of type II collagen [27]. In the early stages of OA, the degradation of predominant proteoglycan, aggrecan, is mainly caused by other proteinases, aggrecanases, such as ‘A Disintegrin and Metalloproteinase with the ThromboSpondin motifs’ (ADAMTS) family [35]. Two members of the ADAMTs family (ADAMT-4 and ADAMTS-5) are also recognized in OA [36]. It has been shown that both enzymes cleave aggrecans by the 2-fold higher prevalence of ADAMTS4 [35]. However, in the animal study involving ADAMTS-4-knockout mice, no significant difference in the progression and severity of OA was observed following surgical induction [37]. Conversely, ADAMTS-5-knockout mice showed a significant reduction in the severity of cartilage destruction compared with wild-type mice [38]. Recent studies have highlighted the contribution of both enzymes in cartilage degradation by individual or combined impact [39]. Although both enzymes seem involved in OA cartilage destruction with prevalence of ADAMTS-5 [34], their contribution still remains questionable (Figs 1 and 2).

For a long time, it was widely accepted that inflammation is absent or weakly present in OA [40]. However, many studies have confirmed the presence of immune cells and proinflammatory cytokines in the synovial tissues of OA patients [41–44]. Why the synovium becomes inflamed in OA remains debatable. The most widely accepted hypothesis is that, once degraded, cartilage fragments fall into the joint and contact the synovium [40]. In contact with foreign bodies, synovial cells react by producing inflammatory mediators, which lead to additional activation of chondrocytes by metalloproteinases and subsequently increased cartilage degradation [40]. Studies have confirmed the up-regulation of interleukin-1-beta (IL-1β) and tumour necrosis factor alpha (TNF-α) [41–45] in OA compared with healthy joints. Intra-articular injection of IL-1β and TNF-α induces proteoglycan loss [46]. The high density of interleukin-1-receptors (IL-1R) in OA cartilage increases the sensitivity of osteoarthritic chondrocytes to this cytokine [47]. Gene therapy utilizing an interleukin-1-receptor antagonist (IL-1R) reduces the expression of collagenase-1 and prevents formation of OA [48] as well as significant reduction in disease progression [49,50]. However, under higher expression of IL-1, the levels of prostaglandin E2 increase [51]. In contrast to IL-1, prostaglandin E2 up-regulates expression of type II collagen and is one of the positive feedback mechanisms to recuperate ECM [52]. Another possible feedback occurs through an increase in bone morphogenetic proteins (BMPs) under the control of cytokines [53,54]. BMPs are growth factors that are members of the TGF-β superfamily that play crucial roles in both chondrogenesis and the induction of proteoglycan synthesis [55,56]. BMPs stimulate both chondrocyte matrix synthesis and terminal differentiation [56]. Chondrocyte terminal differentiation is followed by MMP-13 expression and matrix degeneration [56]. The primary role of the BMPs in OA still remains in question. However, it is well known that one of the main transcription factors (SOX9) in the regulation of mesenchymal chondrogenesis, expression of collagen type II and aggrecan is partly regulated by BMPs and TGF-β [2]. The negative regulator of SOX9 expression is NF-κ B, mainly regulated by TNF-α and IL-1β [57]. Activation of NF-κB and mitogen-activated protein kinase (MAPK) is required for chondrocytes to express MMPs, ADAMTSs and inflammatory cytokines themselves [8,58]. *In vitro* studies suggest that NF-κ B is the upstream inducer of hypoxia-inducible factor (HIF)-2α, which is a transactivatior of hypertrophy chondrocytes genes, including Col10a1, MMP-13 and VEGF [59].

The anabolic–catabolic balance is under the influence of a complex network of signals that regulate tissue homeostasis [2]. Catabolic activity is dramatically increased in the presence of OA [60]. Because of their low rate of turnover, chondrocytes are not able to substitute for loss of ECM [12].

## Stem-cell potential in OA

Stem cells are the foundation cells for every organ, tissue and cell in the body [61]. They may be thought of as a blank microchip that can ultimately be programmed to perform any number of specialized tasks. Moreover, stem cells are self-sustaining and can replicate themselves for long periods of time [61].

Humans originate from the totipotent stem cell, the fertilized egg, through the process of cell proliferation and differentiation [4]. The fertilized egg divides and gives rise to pluripotent embryonic stem cells that can differentiate into any of the three germ layers: ectoderm, mesoderm and endoderm [4]. During embryonic development, stem cells become specialized with loss of self-renewal potential, which makes the pool of terminally differentiated cells with specific functions unable to be renewed [62,63]. There are many specific stem cells referred to ‘adult’ or ‘somatic’ stem cells that are present in adult tissues [62,63]. They are already specialized and produce some or all of the mature cell types within a particular tissue or organ where they reside [62–64].

The ability to obtain cells with proliferation and differentiation potential without sacrificing potential human life is a highly popular and hopeful tool for modern day researchers [62]. Originally, Friedenstein *et al*. discovered that a specified number of fibroblastoid cells isolated from bone marrow have the capacity to form colonies *in vitro* and under appropriate stimulating environmental conditions, small aggregates of bone and cartilage [65,66]. These cells, known as MSC, have the capacity to differentiate into fibroblasts, adipocytes, osteoblasts, chondrocytes and other mesenchymal tissues [67,68]. Although the bearing of these cells in bone marrow has been proven [69,70], further studies confirmed their presence in other tissues. However, even when isolated by density-gradient fractionation, MSCs remain a heterogeneous mixture of cells with varying proliferation and differentiation potentials [71].

It is generally agreed that adult human MSCs do not express the hematopoietic markers CD45, CD34, CD19, CD79a, CD14 or CD11 [67,72] and co-stimulatory molecules CD80, CD86, CD40 or the adhesion molecules CD31 (platelet/endothelial cell adhesion molecule [PECAM]-1), CD18 (leucocyte function-associated antigen-1 [LFA-1]), or CD56 (neuronal cell adhesion molecule-1). They generally express CD105 (SH2), CD73 (SH3/4), CD44, CD90 (Thy-1), CD71 and Stro-1 as well as the adhesion molecules CD106 (vascular cell adhesion molecule [VCAM]-1), CD166 (activated leucocyte cell adhesion molecule [ALCAM]), intercellular adhesion molecule (ICAM)-1, NOTCH3 (neurogenic locus notch homologue protein 3), ITGA11 (Integrin alpha-11) and CD29 [69,70,72–75].

### MSC potential for cartilage repair

Research in cartilage tissue engineering currently focuses on the use of adult MSCs as an alternative to autologous chondrocytes [76]. Studies on cartilage regeneration with adult MSCs demonstrate that bone marrow, adipose and synovial-derived MSCs are most commonly used [4]. Most of these pre-clinical studies have been performed in rabbit models treated with MSCs combined with appropriated scaffold materials and environmental factors [77–80]. Ovine MSCs have been isolated from bone marrow, expanded, characterized and injected with transforming growth factor (TGF) β3 in a fibrin clot [81]. Ovine MSCs have been shown to display the three main characteristics of MSC adherence to plastic, phenotypic profile (positive for CD44, CD105, vimentin and negative for CD34 and CD45), and trilineage differentiation potential [81]. Two months after implantation, histological analysis revealed chondrocyte-like cells surrounded by a hyaline-like cartilaginous matrix that was integrated to host cartilage [81]. Another study showed that a matrix seeded with autologous cells in combination with MSC was able to facilitate regeneration of hyaline-like cartilage [82].

Herein, it is important to note that OA induced by trauma is different from cartilage degradation during pathogenesis of OA. Trauma is one of the causes leading to development of OA. OA generally develops by disturbing the mechanical and biological events that progressively destabilize the balance between synthesis and degradation of cartilage and subchondral bone. Although cartilage degeneration is not homogenous, misbalance in degeneration and synthesis is widely present in OA cartilage at advanced stages when OA is generally diagnosed. Unfortunately, at this stage of the disease, there appears to be little healthy cartilage available. In contrast, experimental OA induced by mechanical trauma represents damaged cartilage surrounded by healthy cartilage tissue. Healthy cartilage may interact differently with MSC construct then damage tissue. For that reason, cell-based cartilage repair has to be performed on the OA experimental model, which is more similar to OA in human. One of the widely supported methods which results in pathological changes resembling those seen in human is OA induction by chemical component, monosodium iodoacetate.

In human studies, autologous bone marrow stromal cells embedded in a collagen gel were transplanted into articular cartilage defects and covered with autologous periosteum [79,80,83–86]. Six weeks after transplantation, arthroscopic and histological grading scores were better in the cell-transplanted group than in the cell-free control group [83]. The defects were filled with a hyaline-like type of cartilage tissue. which stained positively with Safranin-O [86] and clinical symptoms (pain and walking ability) had improved significantly [84]. One year following arthroscopy [79], histological [85] analysis showed that the defects were repaired with fibrocartilage. A comparative study of autologous BMSC *versus* autologous chondrocyte implantation showed that patients younger than 45 scored significantly better than patients older than 45 in the autologus chondrocyte group [87]. However, age did not make a difference in outcome in the BMSC group [87]. This finding may be because of age effects on chondrocyte molecular pathways that are involved in regulation of cell activity [17].

Even though BMSC are commonly used to treat cartilage defects, it is argued that harvesting bone marrow is a painful procedure with donor site morbidity and risk of wound infection and sepsis [4]. For this reason, adipose-derived stem cells (ASCs) obtained from liposuction waste have also been used. Results have confirmed their potential for chondrogenesis, osteogenesis, adipogenesis, myogenesis and some aspects of neurogenesis [88]. Yet, there is no clinically approved cell-based strategy for treatment of OA-based cartilage lesions available for humans yet in Europe.

Chondrogenesis of human adipose-derived stem cells has shown significantly higher expression of chondrogenic markers after 1 week under appropriate conditions [89]. However, a significantly elevated expression of collagen type X, a marker of chondrocyte hypertrophy, was observed after 3 weeks of chondrogenic induction [89]. This indicates that the regulation of cellular activity by growth factors, scaffolds and even gene therapy merits further investigation.

Another potential source for clinical application is synovium-derived stem cells. This was confirmed by comparison of MSC from five different sources; bone marrow, synovium, skeletal muscle, periosteum and adipose tissue [90]. Synovium-derived cells showed high proliferation, chondrogenesis, adipogenesis and osteogenesis potential similar to bone marrow stem cells [91]. Moreover, the pellets derived from synovium were heavier than those from other tissues, because of their higher secretion of cartilage matrix [90,92].

In the animal study, Li *et al*. reported that human MSC-seeded constructs produced better repair of the cartilage defects compared with the chondrocyte-seeded constructs [93]. However, more recently, Tay *et al*. (2012) observed that MSC-seeded constructs regenerated hyaline cartilage-like tissue and restored a smooth cartilage surface, while the chondrocyte-seeded constructs produced mostly fibrocartilage-like tissue with a discontinuous superficial cartilage contour [94].

### MSC regulation

Major potential cartilage regeneration sources involve bone marrow, adipose and synovial tissue [4] with each tissue necessitating a specific isolation procedure [61]. Bone marrow-MSC are directly aspirated into a syringe from bone shafts, whereas adipose-derived MSCs require enzymatic digestion [61]. Subsequently, MSCs proliferate to obtain the cell reservoir [61]. To promote chondrogenic differentiation, the expanded MSCs need to be further cultured in micromass or in scaffold materials, such as polymers, alginate beads, collagen sponges or hydrogels and microspheres [95]. In addition, growth factors loading on MSCs complex enable expression of chondrocyte markers [13,54,96–98]. For hyaline cartilage *in vivo*, hypoxic conditions seem to be the logical choice to stimulate chondrogenesis [99–103]. It has been shown that hypoxia induces expression of crucial genes for cartilage formation like SOX9, SOX6 and SOX5 as well as secretion of ECM molecules typical for hyaline cartilage [13,99–103]. To date, these components provide MSC chondrogenesis, except that this process should be stopped at the pre-hypertrophic stage similar to condrocytes in hyaline cartilage [104]. This is a crucial step because of the different molecular patterns in chondrocytes and hypertrophic chondrocytes, which provide different bio-mechanical characteristics [105].

## Chondroprogenitor cells

### Chondroprogenitor potential

One more potential source of MSC and progenitors for cartilage repair is cartilage itself [106]. Even if the identification, characterization and molecular background of the resident cartilage cells are still quite unknown and unexplored, there is convincing evidence that these cells possess a proliferative and differentiation potential [106–109]. For phenotype identification, these cells are subjected to the procedure of isolation, expansion, identification and differentiation similar to the other cell sources used in cartilage repair [107–110]. They are also more prevalent in OA cartilage than in ‘normal’ cartilage.

Cells isolated from the surface zone of articular cartilage, which exhibit high affinity for fibronectin, possess a high colony-forming efficiency and express the cell fate selector gene Notch-1 and MSC markers, CD105 and CD166 [107–109]. Moreover, these cells have the capacity to differentiate into cartilage in 3-D pellet cultures [111]. These findings indicate that multipotential mesenchymal progenitor cells are present in articular cartilage [108]. Furthermore, chondroprogenitor cells show high telomerase activity and maintenance of telomere length [112]. In the comparison of equine articular cartilage progenitor cells (ACPCs) and bone marrow-derived stromal cells (BMSCs), both sources express cell fate selector gene (Notch-1) and the putative stem-cell markers (Stro-1, CD90, CD166) [110]. However, chondrogenic induction of BMSCs produces hypertrophic cartilage with positive staining for collagen type X. Conversely, collagen type X was not detected in ACPC [110]. Furthermore, the OA cartilage cells were double-positive for CD105 and CD166. Yet, no signs of hypertrophic chondrocytes and osteogenesis were observed in the chondrogenic micromass cultures after 3 weeks [113]. Mesenchymal stem-cell differentiation into hypertrophic cartilage is the major limitation in hyaline functional cartilage production [104]. ACPCs may therefore be considered superior to MSCs from other tissues in cartilage repair [110,113–115]. In studies, cells positive for markers that have been identified in MSC (CD9^+^/CD90^+^/CD166^+^), CD105^+^/CD166^+^ [113] and Notch-1^+^/Stro-1^+^ [114]) were capable of differentiating in chondrocytes and formed cartilage tissue in micromass pellet cultures. These results indicate the opportunity for using OA cartilage as a potential source of cells with cartilage-forming potential. Yet, further investigations are required to explore chondrogenesis regulation *in vitro*.

### Chondrogenesis

Chondrogenesis is a complex process that is initiated by mesenchymal stem cells crowding and condensing on the bone-forming site, followed by maturation into terminally differentiated chondrocytes [116]. This pathway is accompanied by stage-specific ECM production, synchronized by cellular interactions with the matrix, growth and differentiation factors [117]. The latter initiate or suppress cellular signalling pathways and transcription of specific genes in a spatio-temporal manner [117]. Initially, MSCs express adhesion molecules including N-cadherin, N-CAM (Ncam1), tenascin C (Tnc) and versican, which are involved in the compaction and condensation of MSCs regulated by different BMP factors [118]. Through progression of the condensation process, MSCs begin as mesenchymal and condensation markers to express early cartilage markers [Collagen II type (Col 2a1), aggrecan (Agc) and FGF receptor 3 (Fgfr3)] leading to the pre-chondrocyte cell stage of chondrogenesis [119]. Sox 9 is the major transcriptional factor responsible for mesenchymal cell dedication and pre-chondrocyte and chondroblast differentiation [[119], [120]]. It is turned on in chondrogenic/osteogenic mesenchymal cells prior to condensation and remains highly expressed in prechondrocytes/chondroblasts stages and off when the cells undergo pre-hypertrophy [119]. When combined with other transcriptional factors such as Pax/Nkx/Barx2, Sox 9 permits formation of chondrocytes over the osteocyte lineage by negative regulation of Runx2 (Cbfa1) as a domain transcriptional factor required for osteoblast differentiation [[121], [119]]. There are two other Sox family members Sox 5 and Sox 6, co-expressed and regulated by Sox 9, that play a significant role in activation of cartilage-specific genes: type II, IX and XI collagen, aggrecan and cartilage oligomeric matrix protein [[120], [122]]. To reveal the role and spatio-temporal expression of Sox5 and Sox 6, several studies have focused on Sox5; Sox6 single and double null mice. Single gene deletion results in moderate skeletal abnormalities, while double null animals die of severe systemic chondrodysplasia, indicating the importance of the simultaneous function of these two genes [122]. On the other hand, double mutants failed to undergo proper chondroblast differentiation and poorly express essential cartilage ECM components with long delay in initiation of chondroblast proliferation accompanied by general cartilage matrix deficiency [122]. The maintenance of low levels of specific cartilage markers in double mutants is sustained by normal Sox 9 expression [122]. This implies that synchronized action of Sox 5, 6 and 9 trios is required to maintain sufficient ECM component expression and normal matrix composition. Furthermore, these genes, when combined together, are able to suppress expression of hypertrophic and osteogenic differentiation at the same time [119]. Progression through chondrocyte maturation to hypertrophic chondrocytes is repressed by Sox 9 modulation of the Wnt/beta-catenin signalling pathway with beta-catenin degradation or inhibition of beta-catenin transcriptional activity without affecting its stability [123]. In addition, Sox 5 and Sox 6 delay chondrocyte hypertrophy by down-regulating Ihh signalling, Fgfr3, and Runx2 and up-regulating Bmp6 [119]. Further maturation of chondrocytes is essential for the final remodelling of the cartilage into bone. Chondrocytes achieve this maturation through up-regulation of Runx 2, inducing chondrocyte hypertrophy and positive control by BMPs and MMP13 [[124], [125]]. During the transition from pre-hypertrophic to the hypertrophic phase, chondrocyte expression of early chondrogenesis and hyaline ECM components is replaced by collagen X type [119]. Later, hypertrophic and terminal chondrocytes express angiogenic factors, including VEGF, which provide the genesis for vascularization and formation of primary ossification centres within osteoblasts, osteocytes and haematopoietic cells [126]. Equally, terminal chondroytes undergo apoptosis by release of collagen types X and I and mineralization of the ECM [116]. Contrary to growth plate chondrogenesis, normal articular chondrocytes never undergo hypertrophic differentiation, except at the tidemark [119].

## Role of growth factors in cartilage repair

Chondrogenic differentiation of MSCs is induced by various intrinsic and extrinsic factors [71]. Growth factors play the most important role in this process [71]. They represent a group of biologically active polypeptides produced by the body that can stimulate cell proliferation and differentiation [96]. A large number of these growth factors such as the TGF-β superfamily, insulin-like growth factor-1 (IGF-1) and fibroblast growth factor (FGFs) regulate cartilage homoeostasis and integrity as well as its development [4,96].

It has been shown that the TGF-β superfamily plays an important role in promoting chondrocyte proliferation and differentiation [127]. TGF-β1, TGFβ3, BMP-2,-4,-6,-7 are the most examined members of the TGF-β superfamily ([54,97,128,129]. *In vivo* animal studies of cartilage repair showed improved chondrocyte morphology, integration and a much thicker newly formed cartilage layer after treatment with TGF-β1 [128]. Porcine MSCs encapsulated in agarose hydrogels after treatment with TGF-β3 increase the sulphated glycosaminoglycans in surrounding culture media, highlighting their role in cartilage ECM anabolism [97]. Bone morphogenetic proteins are homodimeric molecules that belong to the TGF-β superfamily [96]. Their role is crucial to both chondrogenesis and osteogenesis [96]. Acting synergistically with TGF-β-1 and 3, BMPs induce proteoglycan synthesis in articular cartilage [4,130]. Furthermore, the expression of some BMPs and their membrane receptors is significantly decreased in patients with OA compared with normal human cartilage [55]. This could explain ECM destruction in patients with OA.

Investigative therapy for OA patients using autologus chondrocytes has shown exciting promise after BMP supplementation [131]. The main problem of autologous chondrocyte transplantation therapy is cell differentiation after several passages in cell culture [132]. This change is characterized by an increased expression of type I collagen and a decrease in type II collagen [132]. After BMP-2 loading in 3-D autologus chondrocyte culture, expression of collagen type II was significantly higher [131]. It has been reported that BMP-7 (also known as osteogenic protein-1) has strong anabolic activity in cartilage formation [133].

It has been shown that local delivery of BMP-4 by genetically engineered MDSCs enhanced chondrogenesis in rats [134]. To estimate the duration and effect of transgene expression in rat models, histological and macroscopic observation confirmed the expression of type II collagen 4 weeks after surgery [134]. Moreover, after 24 weeks, animals treated with BMP-4 showed significantly better cartilage repair than untreated animals [134]. It has been shown that TGF-β1 did not provide any additive effect on cartilage repair [134]. Nevertheless, better results were obtained in chondrogenesis of MSC when TGF-β1, IGF-1, BMP-2 and BMP-7 were combined [135].

Fibroblast growth factors are a large family of polypeptide growth factors found in organisms ranging from nematodes to humans [136]. FGF receptors (FGFRs) exist as a gene family of 4 membrane-bound receptor tyrosine kinases (FGFR1-4) that mediate signals of at least 22 fibroblast growth factors (FGF1-22) [137]. FGFs/FGFRs play important roles in multiple biological processes, including mesoderm induction and patterning, cell growth and migration, organ formation and bone growth [137]. Mutations in FGFRs are the aetiology of many craniosynostosis and chondrodysplasia syndromes in humans [138]. The phenotypes of these mutations in animal models have confirmed the role of FGF signalling in both endochondral and intramembranous bone development [138].

FGFR1-3 are expressed during MSC chondrogenesis in embryonic limb development, but not in mature hyaline chondrocytes [139]. The different stages of expression are a potential tool in controlling chondrogenic differentiation [139]. In FGFR3(−/−) MSC culture after loading with FGF-18, type II collagen and proteoglycan decreased, suggesting FGF18 as a selective ligand for FGFR3 [140]. In a rat study of weekly intra-articular cartilage injection for 3 weeks, FGF-18-induced a dose-dependent increase in cartilage thickness of the tibial plateau [140]. Another member of the FGF family frequently cited is FGF-2 [141,142]. An increase in glycosaminoglycan and collagen type II depends on the amount of FGF-2 loaded on MSC culture in chondrogenic medium [141]. Similar to treatment with other growth factors included in stimulation of cartilage repair *in vivo*, FGF-2 has shown promising results in an equine model [142]. Overall, growth factors appear to be one of the main components in improving clinical cartilage regeneration, but they must be precisely combined and loaded on appropriate scaffold materials to simulate the conditions and 3-D structure most similar to the *in vivo* condition.

## Conclusion

Based on self-repair and multilineage potentials, MSCs provide hyaline cartilage regeneration opportunities. Studies on cartilage regeneration with adult MSCs have shown that bone marrow-derived MSCs are the most commonly used cell type to address cartilage regeneration. However, although short-term results appear satisfactory, ‘hypertrophic chondrocyte’ and fibrocartilage formation emerge thereafter with hypertrophically differentiated MSC. Note that fibrocartilage provides a molecular pattern (type I and II collagens, aggrecan, IL-1β and activin-like kinase-1) secreted by hypertrophic chondrocytes, leading to different biomechanical characteristics compared with hyaline cartilage.

Furthermore, harvesting bone marrow is a painful procedure with donor site morbidity and risk of wound infection and sepsis. Hence, both ASCs and synovium-derived stem cells have been considered as alternatives. However, results using these two cell lines have been similar to those obtained employing the bone marrow approach. In fact, although a high expression of chondrogenic markers was initially obtained, they appear to be expressed as collagen type X, confirming the presence of hypertrophy.

Therefore, further investigations into the regulation of cellular activity by growth factors, scaffolds and even gene therapy remain viable options. Recently, one more potential source of MSC and progenitors for cartilage repair engineering from the cartilage itself has been tested. Cells isolated from the surface zone of articular cartilage have the capacity to differentiate into cartilage in 3-D pellet culture. Moreover, no signs of hypertrophic chondrocytes and osteogenesis were observed. Therefore, ACPCs could be considered more adequate than MSCs from other tissues in cartilage repair.
